# The Complex Relationship between Neuromodulators, Circadian Rhythms, and Insomnia in Patients with Obstructive Sleep Apnea

**DOI:** 10.3390/ijms25158469

**Published:** 2024-08-02

**Authors:** Agata Gabryelska, Szymon Turkiewicz, Marta Ditmer, Adrian Gajewski, Dominik Strzelecki, Piotr Białasiewicz, Maciej Chałubiński, Marcin Sochal

**Affiliations:** 1 Department of Sleep Medicine and Metabolic Disorder, Medical University of Lodz, 6/8 Mazowiecka, 92-215 Lodz, Poland; szymon.turkiewicz@stud.umed.lodz.pl (S.T.); marta.ditmer@stud.umed.lodz.pl (M.D.); piotr.bialasiewicz@umed.lodz.pl (P.B.); marcin.sochal@umed.lodz.pl (M.S.); 2 Department of Immunology and Allergy, Medical University of Lodz, 251 Pomorska, 92-213 Lodz, Poland; adrian.gajewski@umed.lodz.pl (A.G.); maciej.chalubinski@umed.lodz.pl (M.C.); 3 Department of Affective and Psychotic Disorders, Medical University of Lodz, 251 Pomorska, 92-213 Lodz, Poland; dominik.strzelecki@umed.lodz.pl

**Keywords:** insomnia, OSA, neurotrophins, BDNF, NGF, NTF, neuromodulation

## Abstract

Obstructive sleep apnea (OSA) has been linked to disruptions in circadian rhythm and neurotrophin (NFT) signaling. This study explored the link between neuromodulators, chronotype, and insomnia in OSA. The participants (n = 166) underwent polysomnography (PSG) before being categorized into either the control or the OSA group. The following questionnaires were completed: Insomnia Severity Index (ISI), Epworth Sleepiness Scale, Chronotype Questionnaire (morningness-eveningness (ME), and subjective amplitude (AM). Blood samples were collected post-PSG for protein level assessment using ELISA kits for brain-derived neurotrophic factor (BDNF), proBDNF, glial-cell-line-derived neurotrophic factor, NFT3, and NFT4. Gene expression was analyzed utilizing qRT-PCR. No significant differences were found in neuromodulator levels between OSA patients and controls. The controls with insomnia exhibited elevated neuromodulator gene expression (*p* < 0.05). In the non-insomnia individuals, BDNF and NTF3 expression was increased in the OSA group compared to controls (*p* = 0.007 for both); there were no significant differences between the insomnia groups. The ISI scores positively correlated with all gene expressions in both groups, except for NTF4 in OSA (R = 0.127, *p* = 0.172). AM and ME were predicting factors for the ISI score and clinically significant insomnia (*p* < 0.05 for both groups). Compromised compensatory mechanisms in OSA may exacerbate insomnia. The correlation between chronotype and NFT expression highlights the role of circadian misalignments in sleep disruptions.

## 1. Introduction

The intricate relationship between obstructive sleep apnea (OSA), insomnia, and neuromodulatory mechanisms presents a fascinating realm of study within the field of sleep medicine. OSA is a prevalent condition characterized by repeated episodes of partial or complete upper airway obstruction during sleep, leading to disrupted sleep architecture and significant daytime morbidity [1,2,3]. The prevalence and impact of OSA on public health are underscored by numerous studies, highlighting its association with cardiovascular disease, metabolic disorders, and cognitive impairment [4,5,6,7,8]. Insomnia, a common comorbidity in OSA patients, exacerbates the patient’s burden by impairing sleep quality and duration, further diminishing quality of life and cognitive function [9,10,11,12].

The neurobiological underpinnings of both OSA and insomnia involve complex interactions among various neuromodulators, including brain-derived neurotrophic factor (BDNF), its precursor proBDNF, glial-cell-line-derived neurotrophic factor (GDNF), neurotrophin-3 (NTF3), and neurotrophin-4 (NTF4) [13,14,15,16,17,18]. These neuromodulators play crucial roles in neural plasticity, resilience, and the regulation of the sleep architecture [17,19,20]. Notably, alterations in the levels of these neurotrophins have been implicated in the pathophysiology of a wide array of neuropsychiatric disorders, including depression [21,22,23,24], Alzheimer’s disease [25,26,27], and vascular dementia [28,29], further emphasizing their importance in maintaining neural health and function. BDNF’s function differs when regulating various neuronal systems. It is necessary to mention that OSA is a highly complex condition, involving both intermittent hypoxia and significant changes in sleep architecture caused by frequent awakenings. Thus, obtaining a proper insight into its pathophysiology requires investigating various factors affecting sleep, such as cortisol and norepinephrine. Studies have demonstrated that the hypothalamus–pituitary–adrenal axis, which regulates cortisol production, might be disrupted in individuals with OSA [30]. Interestingly, norepinephrine uptake inhibitors might decrease the severity of OSA [31]. Both of these factors are related to the circadian rhythm, which may also be somewhat impaired in this condition [32].

Emerging research has begun to elucidate the specific links between OSA and alterations in neuromodulator dynamics. Studies have shown that intermittent hypoxia, a hallmark of OSA, can lead to the dysregulation of BDNF and other neurotrophins, potentially contributing to the cognitive and mood disturbances observed in OSA patients [20,33,34,35]. Peripherally, BDNF might aid in the repair of neurons that maintain airway patency [36]. In the central nervous system, BDNF contributes to breathing regulation via the hypoglossal nucleus [37]. Notably, BDNF’s role differs in its modulation of various neuronal systems. For instance, in the hippocampus, BDNF is vital for neurogenesis and synaptic plasticity, influencing mood regulation and the pathophysiology of depression [38]. The dysregulation of BDNF in this region can precipitate depressive symptoms, while its upregulation is associated with antidepressant effects [38]. In the dopaminergic system, BDNF was shown to affect the activity of dopaminergic neurons and increase dopamine turnover [39]. It also promotes dopamine release in the striatum and hippocampus [40]. In vitro, BDNF was shown to promote the survival of dopaminergic neurons in the midbrain of rat embryos [39]. Interactions between dopamine and BDNF in the mesencephalic neurons might be a crucial process in the pathophysiology of substance addiction [41].

Mutations of the GDNF gene might predispose individuals to OSA independently of other factors, including obesity [42,43]. To date, NTF3 and NFT4 remain poorly researched in the context of OSA. Since NTF4 activates the same receptor as BDNF, it could be suspected that their actions could be redundant; however, this subject requires thorough investigation [44]. NTF3 on the other hand, is considered the most versatile neurotrophin, since it acts upon a wide range of NTF receptors [44]. Apart from their influence on sleep architecture (e.g., the intraventricular administration of NTF3 and 4 was shown to increase the number of NREM episodes [45]) they, similarly to BDNF, might potentially aid in the maintenance of the nerves innervating the upper airway muscle, which vibrations can damage during snoring [20].

Furthermore, insomnia in the context of OSA has been associated with distinct neurotrophin profiles, suggesting a unique neurobiological substrate that may influence treatment responses and outcomes [46,47,48], while chronotype has been shown to be associated with insomnia symptoms among OSA individuals [49].

This study aimed to investigate the relationship between gene expression and protein levels of neuromodulators and circadian rhythm through chronotype and insomnia in OSA patients and explore possible mechanisms involved through a search for predictive factors for insomnia presence and severity.

## 2. Results

No differences were observed between study groups in any of the neuromodulators either on gene expression or protein level. The comparison of demographics, polysomnography, questionnaires, neuromodulator gene expressions, and protein levels between control and OSA groups are presented in Table 1.

Based on the ISI score, the OSA and control groups were divided into subgroups with and without clinically significant insomnia (Ins(+) for ISI ≥ 15 and Ins(−) for ISI < 15, respectively).

Higher gene expression of all neuromodulators was present in Ins(+) in the control group while no differences were observed in the Ins(+) OSA group (all *p* < 0.05). Additionally, BDNF and NFT3 were greater in Ins(−) control compared to Ins(−) OSA (both *p* = 0.007).

In the control subjects, the Ins(+) subgroup scored higher on ISI and ME of CQ (both *p* < 0.001); meanwhile, in the OSA participants, the Ins(+) subgroup exhibited greater results in all questionnaires than Ins(−) (all *p* < 0.05). All comparisons of questionnaire data, neuromodulator gene expressions, and protein levels in the context of clinically significant insomnia are presented in Table 2.

The ISI score correlated with gene expression of all neuromodulators in the control and OSA groups, except for NTF4 (R = 0.127, *p* = 0.172) in the OSA group. On the other hand, the ESS score only positively correlated with the expression of NFT3. Both the ME dimension of chronotype and ESS correlated with the AM dimension within the OSA group, but not the control group. The AM dimension of chronotype was associated with the gene expression of all neuromodulators in the OSA, but none in the control group. No significant relationships were observed between protein levels of evaluated neuromodulators and questionnaire data. All correlations between questionnaire data, neuromodulator gene expressions, and protein levels are presented in Table 3.

The linear regression model for the ISI score in the control group included the following: constant (*p* = 0.509), NTF4 gene expression (*p* < 0.001), ME and AM score of CQ (*p* = 0.006 and *p* = 0.004, respectively), and explained 47.2% of the variance; meanwhile, in OSA, it was comprised of the constant (*p* = 0.209), ME and AM scores of CQ (*p* < 0.001 and *p* = 0.002, respectively), and amounted to 31.4%. All information about the linear regression models is presented in Table 4.

Significant predictors of clinically significant insomnia (ISI ≥ 15) in the control group included the following: NTF3 gene expression (OR: 14.0, 95% CI: 1.3–154.4, *p* = 0.031) and ME and AM score of CQ (OR: 1.3, 95% CI: 1.0–1.5, *p* = 0.017 and OR: 1.3, 95% CI: 1.0–1.6, *p* = 0.049, respectively); meanwhile, in the OSA group, predictors were ME and AM scores of CQ (OR: 1.1, 95% CI: 1.0–1.2, *p* = 0.043 and OR: 1.1, 95% CI: 1.0–1.3, *p* = 0.019, respectively).

## 3. Discussion

In the corpus of the existing literature, investigations concerning the role of neuromodulators in OSA are limited, with the majority of the extant research predominantly concentrating on the protein levels of BDNF. The current study represents a pioneering effort in this domain, to elucidate no significant disparities in the levels of BDNF, proBDNF, GDNF, NTF3, and NTF4 between OSA patients and control subjects, evaluated both at the protein and mRNA expression levels. This finding aligns with the trajectory of contemporary research suggesting that the mere presence of OSA does not intrinsically modulate the systemic concentrations of these neuromodulators [50,51,52,53].

However, the findings of this study elucidate a distinctive pattern in the expression of neuromodulator genes within individuals exhibiting clinically significant insomnia in the control group, marked by an elevated gene expression of all neuromodulators. This pattern was notably absent in the OSA cohort, implying that the underlying pathophysiology of insomnia might entail a compensatory upregulation of neuromodulators in non-OSA individuals, potentially serving as a neuroprotective mechanism to mitigate sleep disruptions [54]. Such compensatory mechanisms appear to be compromised in OSA patients, potentially contributing to the heightened prevalence of depressive disorders and cognitive deficits observed in this demographic [55,56,57,58,59]. This dysregulation may be attributed to the deleterious effects of intermittent hypoxia associated with OSA. For instance, Xie et al. have demonstrated in a murine model that intermittent hypoxia precipitates a downregulation of hippocampal BDNF, correlating with compromised long-term potentiation (LTP) and consequent cognitive impairments [33]. Furthermore, the exogenous administration of BDNF was shown to restore LTP amplitude [33]. This phenomenon aligns with observations from additional animal studies on intermittent hypoxia, which similarly reported a reduction in BDNF levels [60,61,62,63]. However, it must be mentioned that individuals with no clinically significant insomnia had higher expression levels of BDNF and NTF3 in the OSA group compared to controls. This may indicate that the compensatory mechanism is disrupted not by a reduction in the expression of neuromodulators but rather by their constant and increased expression. It may lead to a reduction in the tissue’s sensitivity to their positive neuromodulatory effect and promote the occurrence of clinically significant insomnia [19].

Subsequent analyses revealed a more pronounced association between the ME and AM dimensions of chronotype and neuromodulator gene expression within the OSA cohort. This observation suggests that OSA may modulate or exacerbate the influence of an individual’s chronotype on neuromodulator gene expression, potentially altering sleep architecture and the severity of insomnia as well as presenting a pronounced dysfunction among OSA individuals in the ability to adapt to functioning at various times of the day. Furthermore, the chronotype scores emerged as significant predictors of insomnia severity and the occurrence of clinically significant insomnia across both the control and OSA groups. This phenomenon may reflect the interplay between circadian disruptions induced by OSA and intrinsic circadian rhythms [64,65,66,67]. The influence of chronotype on the timing of sleep and physiological processes, juxtaposed with the sleep–wake cycle disruption characteristic of OSA, could intensify sleep disturbances and modulate neuromodulator levels. The research by Tirassa et al. demonstrated that the chronotype in young healthy women correlates with serum BDNF levels, albeit this correlation was contingent upon the timing of measurements. For instance, individuals of the evening type exhibited an ascension in BDNF levels from morning to evening, whereas morning types displayed a zenith of BDNF levels in the morning [68]. However, this study did not incorporate an assessment of insomnia. Additionally, the evidence suggests an interaction between the circadian clock and BDNF. Specifically, BDNF knockout in zebrafish resulted in the attenuation of rhythmic expression of circadian clock genes [69]. Furthermore, Sadhukhan et al. reported that post-stroke cognitive impairment is associated with the reduced expression of CLOCK and BDNF genes, indicating that transcriptional dysregulation of these genes could underlie cognitive decline post-stroke [70]. Further research is needed to clarify the relationship between the circadian clock and neuromodulators in OSA patients and its possible effect on insomnia. Moreover, it is noteworthy that among genomic factors, only the expression of NTF4 and NTF3 genes in the healthy control group were predictive of insomnia severity and the onset of clinically significant disease, respectively. None of the genes tested were predictors of insomnia in the OSA group.

## 4. Materials and Methods

### 4.1. Participant Recruitment

At the Sleep and Respiratory Disorders Centre in Lodz (Poland), 166 participants were recruited for the study. All individuals were between 18 and 75 years old and had a body-mass index (BMI) of 20–45 kg/m^2^. Psychiatric/neurological/inflammatory/chronic respiratory diseases, infection, cancer, and hypnotic medications were included in the exclusion criteria. The Ethics Committee at the Medical University of Lodz (RNN/432/18/KE) approved the study; all participants provided informed consent.

### 4.2. Polysomnography

Physical examination preceded nocturnal polysomnography (PSG) recording (Alice 6, Phillips-Respironics), which included electroencephalography (EEG), electromyography (EMG), electrooculography (EOG), thermistor gauge, snoring recordings, body position tracking, piezoelectric gauges, and electrocardiogram (ECG) to monitor sleep stages, apnea, hypopnea and arousal events, heart activity, and hemoglobin oxygen saturation (SpO_2_). PSG data were scored according to the American Academy of Sleep Medicine (AASM) guidelines from 2017, Version 2.3, using the epoch length of 30 s [71]. Interpretation was conducted by the same physician-researcher with expertise in PSG evaluation. Apnea was identified as a reduction in airflow to less than 10% of the baseline for a minimum of 10 s. Hypopnea was defined as a decrease in airflow by at least 30% for at least 10 s, along with either a reduction in SpO2 by more than 3% or an arousal. OSA diagnosis and severity was based on the apnea-hypopnea index (AHI) ≥ 5.

### 4.3. Assessment of Gene Expression and Protein Level

In the morning after a PSG examination, blood samples were collected (within 10 min of awakening, around 06:00) using tubes with an EDTA and with clot activator.

Blood samples with the clot activator were immediately centrifuged at 4 °C, and the serum was subsequently collected and stored at −80 °C. The concentration of neurotrophin proteins in the serum was measured using ELISA kits (FineTest for BDNF and proBDNF, EIAab Science for GDNF, NTF3, and NT4, Wuhan, China). Absorbance was read at a wavelength of 450 nm using a BioTek 800 TS absorbance reader (Agilent Technologies, Santa Clara, CA, USA). RNA was isolated from peripheral blood leukocytes using TRIzol reagent (Invitrogen, Waltham, MA, USA), and the RNA Integrity Number (RIN) and concentration were determined using a Nanodrop Colibri Microvolume Spectrometer (Titertek Berthold, Bad Wildbad, Germany). The isolated RNA was reverse transcribed following the manufacturer’s protocol using the SuperScript IV First-Strand Synthesis System (Thermo Fisher Scientific Inc., San Jose, CA, USA). The reverse transcription process included three steps, with annealing conducted at 60 °C for 60 s. Gene expression levels were quantified using a quantitative real-time PCR, with the reaction mixture containing nuclease-free water, Master Mix TaqMan Universal, cDNA, and gene-specific probes (TaqMan assays for BDNF, GDNF, NTF3, and NTF4; reference gene: β-Actin). Each sample and the reference gene were run in triplicate, and the cycle threshold (CT) values were determined. The ΔCt values were calculated and used for mRNA expression analysis using the 2^−∆Ct^ and multiplied by 10.

### 4.4. Questionnaires

In the morning after the PSG examination, the participants completed the following questionnaires: Insomnia Severity Index (ISI), Epworth Sleepiness Scale (ESS), and Chronotype Questionnaire (CQ) comprising two dimensions, morningness-eveningness (ME) and subjective amplitude (AM).

#### 4.4.1. Insomnia Severity Index (ISI)

The severity of insomnia was assessed using a self-report questionnaire. Patients responded to seven questions evaluating various sleep aspects, including difficulties in falling asleep, staying asleep, waking up too early, dissatisfaction with sleep quality, the impact of sleep issues on daily activities, noticeable effects on quality of life, and concerns or distress about sleep. Each question was rated on a scale from 0 to 4 based on its relevance to the patient’s condition. The scores were summed to produce an overall score, which classified patients into four categories: no clinically significant insomnia (0–7), subthreshold insomnia (8–14), moderate clinical insomnia (15–21), and severe clinical insomnia (22–28).

#### 4.4.2. Epworth Sleepiness Scale (ESS)

This questionnaire includes eight questions where patients rated the likelihood of falling asleep in specific situations, such as while sitting and reading, watching TV, or having a conversation. The scale ranges from 0 to 3 for each situation and is designed to evaluate excessive daytime sleepiness. The total score is obtained by summing the responses. A score of 0 to 7 indicates a low probability of abnormal sleepiness, while scores of 8 to 9 reflect an average level of daytime sleepiness. Scores between 10 and 15 suggest potential excessive sleepiness, and scores from 16 to 24 indicate significant daytime sleepiness.

#### 4.4.3. Chronotype Questionnaire (CQ)

The Chronotype Questionnaire is a self-assessment tool used to determine an individual’s chronotype, reflecting their natural preference for activity and sleep times. Participants answer a series of questions regarding their usual sleep–wake patterns, peak periods of alertness, and daily performance. Each response is rated on a scale, and the cumulative score indicates whether the individual is a morning type (AM), evening type (ME), or intermediate type. The AM dimension assesses the preference for morning activities and alertness, while the ME dimension evaluates the tendency towards evening activities and peak performance later in the day. This information helps in understanding a person’s optimal functioning times and aligning daily activities with their natural biological rhythms.

### 4.5. Statistical Analysis

Statistical analysis was performed with SPSS 28.0 (IBM, Armonk, NY, USA). The distribution was evaluated using the Shapiro–Wilk test. Variables with normal distribution were compared using independent Student *t*-tests, and non-normally distributed parameters by Mann–Whitney U test. Nominal variables were analyzed using chi-square tests. Correlations were examined by Spearman’s rank correlation test. Multivariable linear regression with a stepwise procedure was applied to investigate the predictive factors of the ISI score. Logistic regression models were created to search for predictive factors of clinically significant insomnia (ISI ≥ 15) and estimated odds ratio (OR) with 95% confidence intervals (CI). The level of statistical significance was set at *p* < 0.05.

## 5. Conclusions

This comprehensive investigation into the role of neuromodulators in OSA and its association with insomnia severity and chronotype distinctions contributes significantly to the understanding of OSA’s complex pathophysiology. This study’s findings highlight the nuanced interplay between neuromodulator expression, circadian rhythm disruptions, and sleep architecture in OSA patients, diverging from patterns observed in control subjects with insomnia. Notably, the altered regulation of neuromodulators in OSA patients, potentially influenced by chronic intermittent hypoxia, suggests a disrupted compensatory mechanism that might underlie insomnia. Furthermore, the pronounced relationship between chronotype dimensions and neuromodulator gene expression in OSA individuals underscores the potential impact of circadian misalignments on sleep disturbances and neuromodulatory processes. These insights pave the way for future research aimed at elucidating the mechanisms by which OSA affects neuromodulator dynamics and circadian regulation, offering potential targets for therapeutic intervention to ameliorate insomnia and related comorbidities in OSA patients.

## Figures and Tables

**Table 1:** Comparison of demographic, polysomnography, questionnaires, neuromodulator gene expressions, and protein levels between study groups.

		Control (n = 51)	OSA (n = 115)	*p*-Value
Demographic	Age [years old]	45.2 ± 12.4	54.0 ± 11.5	**<0.001**
	Sex (male [n])	33 (64.7%)	98 (85.2%)	**0.003**
	BMI [kg/m^2^]	26.8 (24.3–30.5)	31.6 (27.9–36.2)	**<0.001**
PSG parameters	Sleep Onset Latency [min]	22.5 (10.6–35.9)	21.0 (11.0–40.9)	0.363
	Sleep Maintenance Efficiency [%]	89.7 (82.7–96.7)	86.1 (73.8–93.9)	0.436
	TST [hours]	6.2 ± 1.1	6.2 ± 1.2	0.875
	REM [hours]	1.3 ± 0.6	1.3 ± 0.6	0.939
	nREM [hours]	4.9 ± 0.9	4.9 ± 0.9	0.874
	Arousal Index [events/hour]	10.5 ± 7.9	20.2 ± 14.2	**<0.001**
	AHI [events/hour]	1.8 (1.0–3.1)	26.8 (12.4–46.4)	**<0.001**
	Total number of desaturations	11.5 (6.0–20.8)	146.0 (68.0–293.0)	**<0.001**
	Desaturation Index [events/hour]	2.0 (1.0–3.0)	26.5 (11.6–49.5)	**<0.001**
Questionnaires	ISI score	14.0 (10.3–18.0)	12.0 (9.0–16.0)	0.993
	ESS score	9.0 (5.3–11.0)	8.0 (5.0–12.0)	0.993
	ME score of CQ	23.0 (18.0–27.0)	19.0 (16.0–23.0)	**0.002**
	AM score of CQ	23.0 (18.0–26.0)	21.0 (18.0–24.0)	0.091
Gene Expression	BDNF	4.0 (2.0–13.1)	7.2 (3.2–17.2)	0.065
	GDNF	2.3 (0.6–5.1)	2.6 (1.0–5.3)	0.583
	NTF3	4.6 (1.3–7.8)	4.7 (1.7–11.3)	0.229
	NTF4	5.9 (2.2–11.1)	4.9 (1.7–13.0)	0.874
Protein Level	BDNF [ng/mL]	13.7 (7.5–22.7)	12.0 (6.4–20.1)	0.537
	proBDNF [ng/mL]	5.7 (3.1–10.4)	5.3 (2.8–8.6)	0.485
	GDNF [ng/mL]	94.1 (78.7–116.5)	94.1 (84.3–122.1)	0.577
	NFT3 [ng/mL]	143.1 (120.3–169.6)	131.7 (106.7–156.7)	0.110
	NFT4 [pg/mL]	2.3 (1.5–3.0)	2.2 (1.7–2.7)	0.896

AHI—apnea-hypopnea index; AM—subjective amplitude; BDNF—brain-derived neurotrophic factor; BMI—body mass index; CQ—Chronotype Questionnaire; ESS—Epworth Sleepiness Scale; GDNF—glial-cell-line-derived neurotrophic factor; ISI—Insomnia Severity Index; ME—morningness-eveningness; nREM—non-rapid eye movement; NTF3—neurotrophin 3; NTF4—neurotrophin 4; proBDNF—precursor of BDNF; REM—rapid eye movement; TST—total sleep time. Bold text represents statistically significant results.

**Table 2:** Comparison of questionnaire data, neuromodulator gene expressions, and protein levels in the context of clinically significant insomnia.

		Control		*p*-Value	OSA		*p*-Value	*p*-Value Control vs. OSA ISI < 15	*p*-Value Control vs. OSA ISI ≥ 15
		Ins(−) (n = 29)	Ins(+) (n = 23)		Ins(−) (n = 72)	Ins(+) (n = 46)			
Gene Expression	BDNF	2.7 (1.3–7.1)	8.8 (3.6–21.5)	**0.004**	7.0 (3.0–15.2)	8.4 (3.9–29.5)	0.142	**0.007**	0.809
	GDNF	1.1 (0.4–3.4)	3.7 (1.9–5.5)	**0.012**	2.1 (0.9–4.4)	3.5 (1.2–6.8)	0.085	0.107	0.712
	NTF3	1.8 (0.8–4.9)	7.6 (4.6–16.2)	**<0.001**	4.1 (1.7–10.9)	5.3 (1.78–11.7)	0.487	**0.007**	0.340
	NTF4	5.0 (1.4–7.4)	7.2 (3.4–17.8)	**0.032**	5.5 (2.2–13.0)	4.8 (1.1–13.2)	0.570	0.179	0.112
Protein Level	BDNF [ng/mL]	7.8 (6.1–21.8)	16.8 (10.6–23.8)	0.103	11.4 (6.41–19.9)	12.8 (6.4–21.4)	0.588	0.910	0.296
	proBDNF [ng/mL]	3.9 (2.6–10.6)	6.3 (4.9–10.3)	0.161	5.2 (3.0–8.3)	6.1 (2.6–9.1)	0.788	0.852	0.235
	GDNF [ng/mL]	89.7 (73.1–119.0)	94.6 (86.2–129.9)	0.245	92.5 (83.93–127.5)	96.9 (85.7–116.8)	0.338	0.346	0.739
	NFT3 [ng/mL]	140.5 (119.2–164.3)	155.7 (120.3–173.4)	0.497	133.6 (102.2–156.8)	131.7 (120.3–156.0)	0.666	0.300	0.311
	NFT4 [pg/mL]	1.8 (1.4–2.8)	2.6 (1.8–3.3)	0.113	2.2 (1.8–2.7)	2.24 (1.4–2.8)	0.656	0.239	0.244
Questionnaires	ISI score	11.0 (8.5–13.0)	18.0 (16.0–22.0)	**<0.001**	10.0 (8.0–12.0)	17.0 (15. 8–19.00)	**<0.001**	0.239	0.147
	ESS score	7.0 (3.5–10.5)	9.0 (6.0–12.00)	0.123	7.0 (6.0–12.00)	9.0 (5.8–14.0)	**0.041**	0.904	0.893
	ME score of CQ	20.0 (17.0–23.8)	26.0 (23.0–28.0)	**<0.001**	18.0 (15.0–21.8)	20.5 (17.0–25.0)	**0.015**	0.170	**0.001**
	AM score of CQ	22.5 (17.3–25.8)	25.0 (20.0–26.0)	0.146	19.5 (17.0–22.0)	23.0 (21.0–26.00)	**<0.001**	0.104	0.636

AM—subjective amplitude; BDNF—brain-derived neurotrophic factor; CQ—Chronotype Questionnaire; GDNF—glial-cell-line-derived neurotrophic factor; ISI—Insomnia Severity Index; ME—morningness-eveningness; NTF3—neurotrophin 3; NTF4—neurotrophin 4; OSA—obstructive sleep apnea; proBDNF—precursor of BDNF. Bold text represents statistically significant results.

**Table 3:** Correlations between questionnaire data, neuromodulator gene expressions, and protein levels.

		ISI Score		ESS Score		ME Score of CQ		AM Score of CQ	
		Control	OSA	Control	OSA	Control	OSA	Control	OSA
Questionnaires	ESS score	**R = 0.310** ***p* = 0.025**	**R = 0.208** ***p* = 0.024**						
	ME score of CQ	**R = 0.540** ***p* < 0.001**	**R = 0.352** ***p* < 0.001**	R = 0.137 *p* = 0.339	R = 0.175 *p* = 0.058				
	AM score of CQ	**R = 0.346** ***p* = 0.013**	**R = 0.514** ***p* < 0.001**	R = −0.098 *p* = 0.493	**R = 0.355** ***p* < 0.001**	R = 0.227 *p* = 0.109	**R = 0.228** ***p* = 0.013**		
Gene Expression	BDNF	**R = 0.310** ***p* = 0.025**	**R = 0.223** ***p* = 0.015**	R = −0.219 *p* = 0.119	R = 0.020 *p* = 0.833	R = 0.137 *p* = 0.339	R = 0.049 *p* = 0.597	R = −0.098 *p* = 0.493	**R = 0.251** ***p* = 0.006**
	GDNF	**R = 0.288** ***p* = 0.042**	**R = 0.193** ***p* = 0.038**	R = −0.014 *p* = 0.921	R = 0.008 *p* = 0.934	R = 0.135 *p* = 0.351	R = 0.081 *p* = 0.387	R = −0.048 *p* = 0.741	**R = 0.191** ***p* = 0.039**
	NTF3	**R = 0.347** ***p* = 0.012**	**R = 0.196** ***p* = 0.034**	R = −0.168 *p* = 0.234	**R = 0.268** ***p* = 0.003**	R = 0.158 *p* = 0.269	R = −0.080 *p* = 0.389	R = −0.082 *p* = 0.566	**R = 0.223** ***p* = 0.015**
	NTF4	**R = 0.296** ***p* = 0.033**	R = 0.127 *p* = 0.172	R = −0.074 *p* = 0.603	R = 0.078 *p* = 0.400	R = 0.123 *p* = 0.391	R = 0.058 *p* = 0.535	R = −0.196 *p* = 0.169	**R = 0.225** ***p* = 0.014**
Protein Level	BDNF	R = 0.005 *p* = 0.978	R = 0.097 *p* = 0.470	R = 0.215 *p* = 0.236	R = 0.192 *p* = 0.150	R = 0.033 *p* = 0.859	R = 0.094 *p* = 0.481	R = 0.182 *p* = 0.327	R = −0.097 *p* = 0.470
	proBDNF	R = −0.032 *p* = 0.863	R = 0.142 *p* = 0.285	R = 0.127 *p* = 0.487	R = 0.121 *p* = 0.360	R = 0.042 *p* = 0.823	R = 0.062 *p* = 0.643	R = 0.060 *p* = 0.750	R = −0.041 *p* = 0.760
	GDNF	R = 0.236 *p* = 0.148	R = 0.013 *p* = 0.919	R = 0.082 *p* = 0.620	R = 0.028 *p* = 0.825	R = −0.095 *p* = 0.570	R = 0.203 *p* = 0.105	R = 0.112 *p* = 0.503	R = 0.122 *p* = 0.334
	NFT3	R = 0.147 *p* = 0.373	R = 0.093 *p* = 0.462	R = 0.185 *p* = 0.261	R = 0.071 *p* = 0.576	R = 0.129 *p* = 0.441	R = 0.162 *p* = 0.199	R = 0.125 *p* = 0.453	R = 0.155 *p* = 0.217
	NFT4	R = 0.170 *p* = 0.300	R = 0.092 *p* = 0.466	R = −0.068 *p* = 0.682	R = −0.042 *p* = 0.742	R = −0.055 *p* = 0.742	R = 0.049 *p* = 0.697	R = −0.047 *p* = 0.778	R = −0.087 *p* = 0.489

AM—subjective amplitude; BDNF—brain-derived neurotrophic factor; CQ—Chronotype Questionnaire; ESS—Epworth Sleepiness Scale; GDNF—glial-cell-line-derived neurotrophic factor; ISI—Insomnia Severity Index; ME—morningness-eveningness; NTF3—neurotrophin 3; NTF4—neurotrophin 4; OSA—obstructive sleep apnea; proBDNF—precursor of BDNF. Bold text represents statistically significant results.

**Table 4:** Linear regression models for ISI score in OSA and control group. AM—subjective amplitude; CQ—Chronotype Questionnaire; ISI—Insomnia Severity Index; ME—morningness-eveningness; NTF4—neurotrophin 4; OSA—obstructive sleep apnea.

**Control group**	**ISI Score**				
	**Model**	**R^2^ = 0.472, F = 13.845, *p* < 0.001**			
	**Parameters**	**B**	**B 95% CI**	**t**	***p*-Value**
	**Constant**	**−2.095**	**−8.434–4.244**	**−0.665**	**0.509**
	**ME score of CQ**	**0.317**	**0.095 –0.539**	**2.871**	**0.006**
	**AM score of CQ**	**0.373**	**0.125–0.621**	**3.023**	**0.004**
	**NTF4 gene expression**	**0.719**	**0.359–1.079**	**4.017**	**<0.001**
	**Excluded variables: ESS score, BDNF, GDNF, NTF3 gene expressions**				
**OSA group**	**ISI Score**				
	**Model**	**R^2^ = 0.314, F = 26.047, *p* < 0.001**			
	Parameters	B	B 95% CI	t	*p*-Value
	Constant	−2.766	−7.108–1.575	−1.262	0.209
	**ME score of CQ**	0.476	**0.302–0.649**	5.432	<0.001
	**AM score of CQ**	0.281	**0.106–0.455**	3.193	0.002
	Excluded variables: ESS score, BDNF, GDNF, NTF3, NTF4 gene expressions				

AM—subjective amplitude; CQ—Chronotype Questionnaire; ISI—Insomnia Severity Index; ME—morningness-eveningness; NTF4—neurotrophin 4; OSA—obstructive sleep apnea. Bold text represents statistically significant results.

## Data Availability

Data are available on request from the authors due to privacy restrictions.
